# Atypical herpetic keratitis presenting as multifocal epithelial lesions

**DOI:** 10.7705/biomedica.5518

**Published:** 2020-12-09

**Authors:** Galvis Virgilio, Alejandro Tello

**Affiliations:** 1 Centro Oftalmológico Virgilio Galvis, Floridablanca, Colombia Fundación Oftalmológica de Santander FOSCAL, Floridablanca, Colombia Departmento de Oftalmología, Facultad de Ciencias de la Salud, Universidad Autónoma de Bucaramanga, Bucaramanga, Colombia Universidad Autónoma de Bucaramanga Departmento de Oftalmología Facultad de Ciencias de la Salud Universidad Autónoma de Bucaramanga Bucaramanga Colombia

Humans are the only natural reservoir for herpes simplex virus 1, which can invade its host after contacting mucosal surfaces or through abraded skin. Primary infection, either ocular or extraocular, manifests clinically only in a percentage of people yet to be determined, considered to be less than 20%. Therefore, since approximately 80% of patients are asymptomatic, the infection can spread without being recognized most of the time.

Following primary infection, the virus establishes in sensory neurons where it remains for the lifetime of the host. Upon reactivation, the virus reappears, usually at the mucocutaneous junction of the lips, causing herpes labialis, commonly known as cold sores or fever blisters. However, following reactivation, the virus rarely involves the ophthalmic branch of the trigeminal nerve and causes ocular disease, including keratitis ^1^^-^^6^.

A 60-year-old woman presented with red eye, a foreign body sensation, and moderate photophobia in the right eye for one week. She was receiving a combination of antibiotics and topical steroids prescribed by a general practitioner.

**Figure 1:**
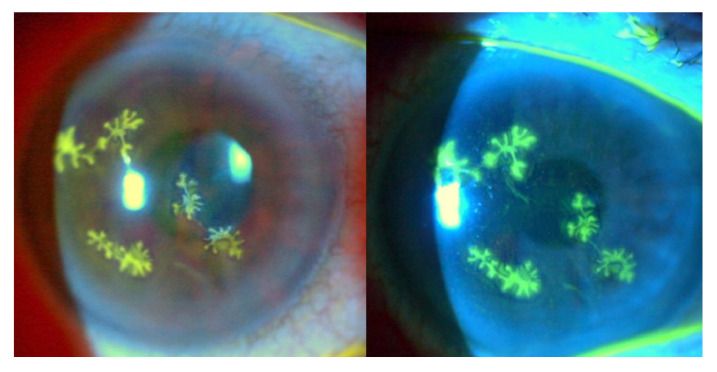
Multiple dendritic epithelial ulcers stained with fluorescein in a 60-year-old woman who received topical steroids prescribed by a general practitioner. Clinical photographs in a slit lamp illuminated with white light (left) and using a cobalt blue filter (right)

Upon examination in the slit lamp, four separate dendritic-shaped epithelial lesions highly suggestive of epithelial herpetic keratitis were found. However, the lesions presented with a multifocal compromise, which is a very uncommon presentation since these corneal epithelial ulcers are usually single lesions of variable size (figure 1). One factor that could have led to this atypical presentation was the use of topical dexamethasone. Since the 1960s, it has been considered that steroids are contraindicated when there is corneal epithelial compromise caused by herpes simplex virus ^7^^-^^11^ and that the use of steroids in epithelial herpetic keratitis increases the risk of the ulcer progressing from a dendritic ulcer to a geographical ulcer and the possibility of multifocality ^10^^,^^11^.

This case reaffirms the probability that the use of these substances can worsen the clinical picture when the virus is compromising the epithelium. Therefore, since this condition can be confused with infectious conjunctivitis, general practitioners should be cautious and look for symptoms such as photophobia, which would suggest corneal involvement. The use of topical steroids should be avoided, since these drugs can aggravate the ocular affectation caused by the herpes virus, as in this case.

Keywords: Keratitis; herpetic eye disease; herpes simplex; cornea; corticosteroids.

Palabras clave: queratitis, enfermedad ocular herpética, herpes simplex, córnea, corticosteroides.
